# Robot-Assisted Image-Guided Interventions

**DOI:** 10.3389/frobt.2021.664622

**Published:** 2021-07-12

**Authors:** Michael Unger, Johann Berger, Andreas Melzer

**Affiliations:** ^1^ Innovation Center Computer Assisted Surgery, Leipzig, Germany; ^2^ Institute for Medical Science and Technology, IMSaT, University Dundee, Dundee, United Kingdom

**Keywords:** Imaging, robotics, image-guided interventions, robot assistance, CT, US, MRI

## Abstract

Image guidance is a common methodology of minimally invasive procedures. Depending on the type of intervention, various imaging modalities are available. Common imaging modalities are computed tomography, magnetic resonance tomography, and ultrasound. Robotic systems have been developed to enable and improve the procedures using these imaging techniques. Spatial and technological constraints limit the development of versatile robotic systems. This paper offers a brief overview of the developments of robotic systems for image-guided interventions since 2015 and includes samples of our current research in this field.

## Introduction

The main benefits of image-guided procedures in comparison to open or endoscopic surgery are reduced invasiveness and avoiding general anesthesia. The usage of computer-based systems to provide pre- and intra-operative imaging to perform minimally or non-invasive interventions has become the standard procedure in many medical fields. Over the years, technological advances lead to improvements of existing imaging modalities as well as the emerging of new ones each offering different characteristics (Alam et al., 2018; Zaffino et al., 2020). The most common imaging modalities used to acquire the necessary pre-operative images are computed tomography (CT) and magnetic resonance tomography (MRI), due to their high spatial resolution and versatility. The utilization of ultrasound imaging (US), furthermore, allows for real-time intra-operative imaging due to its fast image acquisition. Much research has been conducted over the last 20 years to optimize image-guided interventions and enhance the cost-effectiveness and general treatment outcome (Cleary and Peters, 2010). The introduction of these procedures into the clinical domain, however also leads to the necessity of adapting to constrained working environments. Due to the missing direct sight into the situs, the surgeons have to ensure very high precision in handling the surgical tools, while relying on image data and in most cases tracked navigation systems. To provide optimal assistance, the development of actuated manipulators and robotic systems received much attention. By co-registering these systems with medical imaging, it is possible to achieve higher accuracy and to alleviate highly complex procedures compared to manual performance.

Since the 2000 s research has aimed for advancement in imaging-robotics to develop novel assistance systems. In 2006 *Cleary et al.* reported on the development of several new devices to support interventions under CT-, MRI- and US-guidance (Cleary et al., 2006). Interventional robots like the *AcuBot* (Stoianovici et al., 2003), the *B-Rob* (Kronreif et al., 2003), or the *INNOMOTION* (Melzer et al., 2008) proved early on that robots can provide high accuracy and repeatability, especially for the placement and steering of interventional cannulae and was the first MRCT robot with CE Mark approval. However, due to the complex development process, robotic systems have long development cycles. This review offers a brief overview of the current directions of robotic systems for image-guided interventions in recent years. The published literature since 2015 was analyzed and the advances in the fields of CT-, MR-, and US-guided interventions were investigated. Robotic systems for image-guided interventions are briefly described and discussed. The focus was set to the latest advances in academia and applied research on commercial devices. Developments to improve specific components like user interfaces, user experience, haptics, etc. were not included.

## CT-Guided Interventions

The high spatial resolution of computed tomography (CT), especially of structures with a high density such as bone, makes this method one of the major imaging modalities in medicine. It is commonly used in head and neck, as well as lung interventions, for angiography and imaging of the axial skeleton and extremities. The fast image acquisition makes it a useful tool for intraoperative imaging. However, since CT imaging involves significant exposure of ionizing radiation for both patient and physician, the ratio of clinical benefit and risk must be taken into account. Therefore, in the last years, much effort has been made to introduce robotic systems that can operate under CT guidance, to alleviate the radiation exposition.

The positioning and insertion of needles and cannulae to perform biopsies or therapeutic procedures (e.g., thermal ablation) is the prominent use case for CT-guided robotics and regular enhancements for such systems are still provided. In 2015 *Cornelis* et al. introduced a study on the *MAXIO* robot (Perfint Healthcare Pvt. Ltd., India) to compare fluoroscopy-guided manual and CT-guided robot interventions in a porcine model (Cornelis et al., 2015). The system comprises an articulated arm to perform motion along three axes and a steerable needle guide, movable along two additional axes. It was identified to reduce the number of confirmatory scans and the need for needle manipulations. In 2018 *Smakic* et al. published confirmatory results for out-of-plane CT-guided interventions (Smakic et al., 2018). Similarly, *Hiraki* et al. evaluated the *Zerobot* (Okayama University, Japan), consisting of a mobile mounted manipulator arm with 6 degrees of freedom and attachable needle holder (Hiraki et al., 2017). This system is designed for use in sliding gantry CT scanners and can, therefore, not fit into most conventional CT devices. With a novel master-slave manipulator Won et al. introduced an alternative principle to remotely perform biopsies and radiofrequency ablations (Won et al., 2017). The end-effector for needle insertion is attached to a five-axis robotic arm, mounted on a mobile cart and optical tracking is used to reference the system with the CT space.

Despite the reported benefits of these systems, operations with steerable arms are restricted by the limited space inside the CT bore. With the aim to enhance flexibility and reachable workspace, approaches were, made to design and test table or body mounted robots. *Yang* et al. designed a system for lung biopsies, consisting of two modules (Yang et al., 2017). One component with four degrees of freedom is mounted on the patient table and a second module with three additional degrees of freedom that is supporting tendon–sheath transmission performs needle orientation and automated insertion. *Ben-David* et al. performed a study with a similar prototypical body-mounted system developed by *XACT Robotics, Ltd.* (Caesaria, Israel) (Ben-David et al., 2018). *Shahriari* et al. tried to further enhance precision and image control by developing a table-mounted arm, that utilizes CT image fusion with electromagnetic sensor data (Shahriari et al., 2017). This allows for remote steering with a real-time electromagnetic tracker.

Besides needle positioning, robotics CT-guided spine surgery and orthopedics have been developed in recent years. As per *Feng* et al. the *TiRobot* system (TINAVI Medical Technology Co., Ltd., China) aims to achieve a more effective treatment outcome for the insertion of pedicle screws compared to the typical fluoroscopy-assisted freehand procedure (Feng et al., 2019). This system comprises a robotic arm with 6 degrees of freedom mounted on a mobile platform and utilizes intraoperative C-arm images. As an alternative *O’Connor* et al. presented the *MAZOR X* platform, consisting of a workstation for surgical planning and a separate manipulator that is attachable to Jackson table bedframes (O’Connor et al., 2021). *Khan* et al. judged this system to decrease treatment and fluoroscopy time and, thereby, reduce radiation exposure (Khan et al., 2019). Robotic assistance, furthermore, improves the efficiency in knee arthroplasty interventions. *Marchand* et al. reported on increased accuracy and significantly reduced flexion and extension gaps, using the *MAKO* robot (Stryker, United States) (Marchand et al., 2019). Another system worth mentioning in this context is the mobile mounted *ROSA ONE* ^*®*^ robotic arm (Zimmer Biomet, United States) that can be used in various interventions of brain and spine surgery. *Carai* et al. successfully utilized this system for diffuse intrinsic pontine glioma (DPIG) stereotactic needle biopsies in single session scenarios (Carai et al., 2017) and it assisted in the first deep brain stimulation with an FDA-approved robot (Vadera et al., 2017). The *ROSA* device provides high accuracy in placing pedicle screws, as shown by Lefranc and Peltier (2015), and performing circumferential arthrodesis, as presented by Chenin et al. (2016).

By providing high precision, speeding up interventions and enabling the user to operate remotely from outside the CT-room, utilizing such robotic systems could reduce complications and radiation exposure significantly. However, the necessity of pre- or intraoperative CT imaging still implies radiation exposure to a significant extend and the need for a reduction of the effective dose remains. *Hunsche* et al. published their research on radiation reduction in stereotactic neurosurgery (Hunsche et al., 2017). They reported the potential of a low dose reference method by using 2D 3D intensity-based registration for lead localization in deep brain stimulation under robotic assistance with the *ROSA* robot. *Sensakovic* et al. tackled the need for radiation reduction as well by introducing an alternative low-dose CT protocol for robot-assisted pediatric spinal surgery using the *MAZOR* system (Sensakovic et al., 2017). Besides the efforts of optimizing the CT-imaging modality to reduce radiation, the growing possibilities to utilize other imaging methods like magnetic resonance imaging (MRI) as a radiation-free alternative must be considered. *Hungr* et al., designed and evaluated the second version of their *LPR* (light puncture robot) for thoracic and abdominopelvic interventional radiology procedures. This system can be operated under CT and MRI guidance (Hungr et al., 2016). It is mounted on the patient and operates with MRI-compatible ultrasonic motors. *Spyrantis* et al. also assessed the possibility to use preoperative MRI instead of CT, to perform stereo-electroencephalography with the *ROSA* robot and reported radiation reduction and safe performance (Spyrantis et al., 2018). *Lonner* et al. even went as far as to design an image-free handheld robotic system for bone resection (Lonner et al., 2015). *Ponzio* et al. tested the *Navio* device against the *MAKO* robot in a comparative study and reported sufficient accuracy and the disadvantages of a preoperative CT-scan in presence of such alternative methods (Ponzio and Lonner, 2015).

## MR-Guided Interventions

Magnet resonance imaging (MRI) enables image-guided interventions without exposing the patient to ionizing radiation. MR technology provides arbitrary slice position, 3D images with variable soft-tissue contrast at near real-time speed. While MR imaging provides high-quality visual information during interventions, it has limitations for conventional as well as robot-assisted interventions. The spatial constraints inside the MRI bore limit the access to the patient during imaging. This also hinders utilizing robotic systems, as these need to fit in the residual space between the patient and the MRI. Furthermore, the high-strength magnetic field impedes the usage of conventional, metal-based materials for the robotic systems. To avoid degrading the image-quality, special considerations need to be taken when designing robotic systems for MR-guided interventions.

### Robotic Systems for MR-Guided Interventions

MR image-guided robot-assisted interventions i.e., cannula placement for biopsies, allow for precise positioning of the probe while ensuring tissue classification. The robot carries the potential to speed up the procedure thereby reducing the burden of the patient lying uncomfortably in the MRI (5). He et al. developed a body-mounted robot with fluid-driven actuators for the positioning of a needle guide (He et al., 2020). The system requires manual placement of the robot on the patient and guided manual coarse targeting based on an initial MR dataset. The needle placement for biopsy or RF ablation is performed manually by the interventionalist out-of-bore. Marker-based tracking was used to measure and position the needle holder pose. Patel et al. proposed a body-mounted robot for needle-based percutaneous interventions (Patel et al., 2021). The system uses piezoelectric motors to align the needle guide with the planned insertion trajectory using MR imaging. Li et al. developed a body-mounted robot for lumbar spinal injections under MR guidance (Li et al., 2020). The system uses piezoelectric actuators to position the cannula guide. Fiducial markers are used to register the robot to the image space. In a manual work step, the cannula is inserted, the placement is verified, and the contrast agent is injected.

Moreira et al. showed a parallel robot for prostate biopsies (Moreira et al., 2016). The system is capable of needle tracking and steering, therefore, is capable of reaching targets behind obstacles. A piezoelectric actuator was used to automatically drive the needle to the target. Chan et al. described a custom-made robot for MR-guided breast biopsies (Chan et al., 2016). The system uses piezoelectric motors to align the needle path with the target. Hata et al. proposed a robotic instrument guide for orienting cryotherapy probes for the treatment of renal cancer (Hata et al., 2016). The robot is body-mounted and comprises two piezoelectric ring-shaped actuators. Fiducial markers attached to the instrument guide were used for the automatic registration. The instrument must be manually positioned using the guide. The correct instrument placement needs to be confirmed with multi-slice images.

MR-guided catheter-based interventions are an emerging field due to the advances in real-time MR-imaging and catheter tracking. The live-imaging enables faster procedures while allowing to monitor the instrument’s position. The interventionalist needs to interact with the MR-scanner while performing the procedure. Thus, the controls and monitors need to be brought into the MR-suite, or the procedure has to be performed from the control room. Therefore, Lee et al. developed a robotic system for cardiac electrophysiological interventions (Lee et al., 2018). The system is based on a hydraulic master-slave approach. The operator controls and actuation units are located in the control room and the motions are transmitted *via* hydraulic tubes through the waveguide. The catheter robot is MR-safe according to ASTM F2503-13. A standard EP catheter can be used with the system enabling passive, semi-active, or active tracking. Kundrat et al. showed a robotic platform for endovascular procedures (Kundrat et al., 2021). The system is based on a pneumatic master-slave approach. Equivalent to Lee et al., the master device comprising the operator controls is located in the control room and pneumatic tubes transmit the operator’s motions through the waveguide. The slave device enables the manipulation of both catheter and guidewire in two degrees of freedom (feeding/retracting + rotation).

## Robotic-Assisted US-Guidance

In contrast to MR- and CT-imaging, ultrasound imaging is widely available, because of its low cost and non-invasive imaging technique. Due to its properties, US imaging requires maintaining contact with the patient throughout the intervention. To ensure precise and reproducible imaging, robotic-assistance has been investigated to improve the imaging process or enable new intervention techniques.

During manual US imaging, the interventionalist needs to provide the dexterity and sensitivity to position the US probe while incorporating the anatomical conditions. Thus, complex technical systems are necessary to automate the imaging process and also ensure patient safety.

Lindenroth et al. designed a soft end-effector to attach a common US probe (Lindenroth et al., 2017). The end-effector consists of three linear fluid-driven actuators providing three translational and three rotational degrees of freedom. The used soft actuators enable passive compliance with human robot interaction as excessive force is deflected away from the patient. While this approach eases the system design from a safety point-of-view, the deformation of the end-effector inhibits knowing the exact position of the ultrasound probe. Therefore, the system is limited for the acquisition of US images, but can’t be easily used for providing image-guidance.

Active safety can be achieved through additional sensors. Huang et al. developed a robotic arm-based system holding a standard US probe to acquire a 3D ultrasound volume (Huang et al., 2019). A depth camera (Kinect, Microsoft Corporation, United States) was used to reconstruct the skin surface. The information on the surface normal-vectors was used to follow the surface and reposition the probe accordingly.

Virga et al. developed a system for the screening of abdominal aortic aneurysms (Virga et al., 2016). A depth camera (Kinect, Microsoft Corporation, United States) was used to register the patient to the robotic system (LBR iiwa, Kuka AG, Germany) and estimate the position of the skin surface. An MRI atlas was used to calculate the trajectory of the robot and follow the aorta. The system was refined by Hennersperger et al. for the automatic acquisition of 3D ultrasound volumes (Hennersperger et al., 2017). Based on a CT or MR dataset and the registration between the robot, the phantom, and the image dataset, the system can be used to plan a trajectory that the robot follows autonomously.

Seitz et al. developed a robotic system for USgRT (Seitz et al., 2020). The system is capable to compensate the motion of the patient, for example breathing. Therefore, the torque sensors integrated into the robotic arm (LBR iiwa, Kuka AG, Germany) were used to control the applied force.

Berger et al. developed a system utilizing two robotic arms (LBR iiwa, Kuka AG, Germany) for US-guided interventions (Berger et al., 2018). One arm can be used in hand-guiding mode to position a mobile US probe (Clarius L7, Clarius Inc., Canada) to plan an intervention. The second arm will position a therapeutic instrument, e.g., needle holder or FUS transducer, according to the planned trajectory. The system was further improved to automatically track anatomical structures using US-guidance (Unger et al., 2020). By automatically segmenting a targeted vessel, the system was able to follow the trajectory of that vessel while maintaining the coupling between the ultrasound probe and the skin surface.

Low-cost robotic arms may not provide internal sensors to measure the forces applied to the system. To enable safety-compliant human robot interaction, such systems need to be outfitted with additional sensors. Mathiassen et al. developed a robotic system based on an industrial robotic arm (UR5, Universal Robots, Denmark) with a force-torque sensor attached to the end-effector holding a standard US probe (Mathiassen et al., 2016). Control schemes were designed and implemented to achieve force control. The system used a haptic device (Phantom Omni, 3D Systems, Inc., United States) to provide telemanipulation with force feedback capabilities.

## Discussion

Robotic systems for image-guided interventions have been published to a large extend. The high precision and reproducibility of robot-assistance enables a wide range of applications for image-guided therapies. Specific requirements for each imaging technique led to the development of use case-specific devices and systems. Table 1 summarizes the systems reported in this work.

**TABLE 1:** Overview of the reported systems.

Imaging modality	Intented clinical use	Ref	System name/vendor	Characteristics
CT	Needle guidance in liver	Cornelis et al. (2015)	MAXIO/Perfint Healthcare Pvt. Ltd., India	Combination of articulated arm and attached steerable needle guide
	Percutaneous diagnostics and therapeutics	Smakic et al. (2018)		
	Needle guidance	Hiraki et al. (2017)	Zerobot/Okayama University, Japan	Mobile mounted, designed for sliding gantry (does not fit in most conventional CT scanners)
	Needle guidance for abdominal biopsies and ablations	Won et al. (2017)	−/−	Mobile mounted arm; co-registered with optical tracking, remotely steered
	Needle guidance for lung biopsy	Yang et al. (2017)	−/−	2 modules; tendon sheath transmission, automated insertion
	Needle guidance in percutaneous interventions	Ben-David et al. (2018)	-/XACT robotics Ltd., caesaria, Israel	Body mounted prototype; based on (10)
	Needle guidance	Shahriari et al. (2017)	−/−	table mounted arm, CT-image fusion with EM sensor data, remote steerng with EM tracker
	Pedicle screw insertion in spine surgery and othopedics	Feng et al. (2019)	TiRobot/TINAVI medical technology Co., Ltd., China	Mounted on mobile platform, based on intraoperative C-arm imaging
	Pedicle screw insertion in spine surgery and othopedics	O’Connor et al. (2021)	Mazor X Stealth™/Medtronic, United States	Attachable to jackson table bedframes, additional side arm for sterile screen tablet
	Pediatric spinal surgery	Sensakovic et al. (2017)		
		Khan et al. (2019)		
	Knee arthroplasty	Marchand et al. (2019)	MAKO/Stryker, United States	Mobile mounted arm; supports multiple use cases for total hip and knee replacement
	(DPIG) stereotactic needle biopsies, deep brain stimulation, pedicle screw insertion, circumferential arthrodesis	Carai et al. (2017)	ROSA ONE^®^/Zimmer biomet, United States	Supports multiple use cases for spine and brain surgery
		Vadera et al. (2017)		
		Lefranc et al. (2015)		
		Chenin et al. (2016)		
		Hunsche et al. (2017)		
	sEEG electrodes implantation	Spyrantis et al. (2018)		
	Knee arthroplasty	Lonner et al. (2015)	Navio/Smith & nephew, United States	Handheld robotic device
		Ponzio et al. (2015)		
CT/MRI	Thoracic and abdominopelvic interventional radiology procedures	Hungr et al. (2016)	LPR (light puncture robot)/	Body mounted; piezoelectric motors + bowden cables
MRI	Needle guide	He et al. (2020)	−/−	Body mounted; fluid-driven actuators; marker-based tracking
				Manual coarse targeting based on an initial MR dataset; manual needle positioning out-of-bore
	Needle guide (Shoulder Arthrography)	Patel et al. (2021)	−/−	Body mounted; piezoelectric motors
	Needle guide (lumbar spinal injections)	Li et al. (2020)	−/−	Body mounted; piezoelectric motors; Fiducial markers
	Prostate biopsies	Moreira et al. (2016)	MIRIAM Robot	table-mounted parallel robot; piezoelectric motors; needle tracking and steering; automatic needle insertion
	Breast biopsies	Chan et al. (2016)	−/−	table-mounted parallel robot; piezoelectric motors; manual needle insertion out-of-bore
	Needle guide (cryotherapy of renal cancer)	Hata et al. (2016)	−/−	Body mounted; piezoelectric motors
	Intracardiac catheterization	Lee et al. (2018)	−/−	table-mounted parallel robot; electro-hydraulic actuators; tele manipulator
	Endovascular instrumentation	Kundrat. et al	−/−	table-mounted; electro-pneumatic actuators; tele manipulator
US	-	Lindenroth et al. (2017)	−/−	Soft end-effector; fluid-driven actuators; passive safety
	3D US acquisition	Huang et al. (2019)	Epson C4-A601S/Seiko	Depth camera; surface reconstruction; automatic probe positioning
			Epson corporation, Japan	
	Screening of abdominal aortic aneurysms	Virga et al. (2016)	LBR iiwa/Kuka AG, Germany	Depth camera; surface reconstruction; automatic probe positioning based on skin surface and MRI data
	3D US acquisition	Hennersperger et al. (2017)	LBR iiwa/Kuka AG, Germany	
	US-guided RT	Seitz et al. (2020)	LBR iiwa/Kuka AG, Germany	Optical tracking; breathing compensation
	US-guided biopsies/FUS	Berger et al. (2018)	LBR iiwa/Kuka AG, Germany	Dual-arm-system
	US-guided FUS	Unger et al. (2020)	LBR iiwa/Kuka AG, Germany	Vessel following; impedance mode
	US-imaging	Mathiassen et al. (2016)	UR5/Universal robots, Denmark	Force-torque sensor; haptic device for telemanipulation

In CT-guided interventions, robotic systems are mandatory to prevent unnecessary radiation exposure while performing precise image-guided interventions. Although MR-imaging is used more widely, the reduced scanning time still favors CT imaging. The provided literature shows that CT-guided systems remain under constant advancement and new devices are still emerging. However, these systems are still use-case-specific and the research of more versatile robots should be promoted to improve flexibility and acceptance in the clinic. The ROSA system provides a first successful solution to perform procedures in different use-cases for brain and spine surgery (Lefranc and Peltier, 2015; Chenin et al., 2016; Carai et al., 2017; Vadera et al., 2017).

In the field of MR-guided robotic interventions, few advances were made. Due to the complex process of the design and development of MR-compatible systems, use-case-specific robotic systems are proposed. Material available and spatial constraints are given by the MR scanner limit the development of a versatile and robust robot. Thus, even for a class of use-cases like needle and instrument placement, special procedure-specific actuators are developed. Body mounted robots may enable using the same robot for different procedure types, but currently, body-mounted robots provide only an instrument guide and the instrument placement process is still manual labor. Another key benefit of body-mounted robots is that only organ displacement under the skin surface needs to be compensated. The next best solution is table-mounted robots (Melzer et al., 2008) but those have to compensate overall patient movements. Most challenging are robots not attached to both MRI or CRT because the table movements need to be taken into consideration (Cornelis et al., 2015).

In the case of catheter interventions, the currently available robotic systems enable teleoperation rather than automated procedures. Hereby, real-time MR imaging is required to provide visual feedback to the user. Conventional diagnostic MRI systems are not designed for interventional imaging. Although fast imaging is provided real-time image guidance is not yet commercially available. A key requirement for MRI-guided interventions is a low latency for clinical decision-making (Campbell-Washburn et al., 2017). The latency is impacted by the imaging as well as the reconstruction. The imaging can be accelerated by optimizing the sequences for a specific procedure. The reconstruction process is continuously sped up by improving the efficiency of the used algorithms (Frahm et al., 2019) but more powerful machines are needed to run complex image guidance schemes.

Robotic assistance for US imaging demands very dexterous and sensitive systems to enable direct human robot interaction. Robot-assisted systems enable automated, repeatable image acquisition. Safety compliance can either be achieved passively by using soft end-effectors or actively by measuring and controlling applied forces. A disadvantage of using soft end-effectors is the loss in precision. Most of the researchers use sensors, either internal or subsequently added ones, to limit the force applied to the patient. Using depth cameras, systems for the automatic registration to MRI or CT images, and following along the surface contour of the patient.

In conclusion, robotic systems for image-guided interventions are widely investigated. Due to the high demand in regards to the operator and patient safety and the resulting high system complexity, few approaches make it from early investigations to a clinical system (Melzer et al., 2008). But as the field progresses, the gained understanding will to available systems and, therefore, help improve the overall treatment capabilities. Among the current challenges is the additional time and effort needed to deploy robotic systems for interventions. This additional effort must be met by better outcomes to overcome restraints in utilizing robotic assistance. But the limited availability of clinical systems impedes broad investigations assessing the impact and benefits of robot-assisted interventions. Furthermore, these interventions are conceptionally different from classic routines. Users are confronted with new complex systems and novel devices need to provide ease of use to push the acceptance of robotic systems.

## References

[B1] Alam I. S. Steinberg I. Vermesh O. van den Berg N. S. Rosenthal E. L. van Dam G. M. (2018). Emerging Intraoperative Imaging Modalities to Improve Surgical Precision. Mol. Imaging Biol. 20, 705–715. 10.1007/s11307-018-1227-6 29916118

[B2] Ben-David E. Shochat M. Roth I. Nissenbaum I. Sosna J. Goldberg S. N. (2018). Evaluation of a CT-Guided Robotic System for Precise Percutaneous Needle Insertion. J. Vasc. Interv. Radiol. 29, 1440–1446. 10.1016/j.jvir.2018.01.002 29628297

[B3] Berger J. Unger M. Landgraf L. Bieck R. Neumuth T. Melzer A. (2018). Assessment of Natural User Interactions for Robot-Assisted Interventions. Current Directions in Biomedical Engineering 4, 165-168. 10.1515/cdbme-2018-0041

[B4] Campbell-Washburn A. E. Tavallaei M. A. Pop M. Grant E. K. Chubb H. Rhode K. (2017). Real-time MRI Guidance of Cardiac Interventions. J. Magn. Reson. Imaging 46, 935–950. 10.1002/jmri.25749 28493526 PMC5675556

[B5] Carai A. Mastronuzzi A. De Benedictis A. Messina R. Cacchione A. Miele E. (2017). Robot-Assisted Stereotactic Biopsy of Diffuse Intrinsic Pontine Glioma: A Single-Center Experience. World Neurosurg. 101, 584–588. 10.1016/j.wneu.2017.02.088 28254596

[B6] Chan K. G. Fielding T. Anvari M. (2016). An Image-Guided Automated Robot for MRI Breast Biopsy. Int. J. Med. Robotics Comput. Assist. Surg. 12, 461–477. 10.1002/rcs.1760 27402476

[B7] Chenin L. Peltier J. Lefranc M. (2016). Minimally Invasive Transforaminal Lumbar Interbody Fusion with the ROSATM Spine Robot and Intraoperative Flat-Panel CT Guidance. Acta Neurochir 158, 1125–1128. 10.1007/s00701-016-2799-z 27068043

[B8] Cleary K. Melzer A. Watson V. Kronreif G. Stoianovici D. (2006). Interventional Robotic Systems: Applications and Technology State‐of‐the‐art. Minimally Invasive Ther. Allied Tech. 15, 101–113. 10.1080/13645700600674179 PMC3107540 16754193

[B9] Cleary K. Peters T. M. (2010). Image-Guided Interventions: Technology Review and Clinical Applications. Annu. Rev. Biomed. Eng. 12, 119–142. 10.1146/annurev-bioeng-070909-105249 20415592

[B10] Cornelis F. Takaki H. Laskhmanan M. Durack J. C. Erinjeri J. P. Getrajdman G. I. (2015). Comparison of CT Fluoroscopy-Guided Manual and CT-Guided Robotic Positioning System for *In Vivo* Needle Placements in Swine Liver. Cardiovasc. Intervent Radiol. 38, 1252–1260. 10.1007/s00270-014-1016-9 25376924 PMC5482420

[B11] Feng S. Tian W. Sun Y. Liu Y. Wei Y. (2019). Effect of Robot-Assisted Surgery on Lumbar Pedicle Screw Internal Fixation in Patients with Osteoporosis. World Neurosurg. 125, e1057–e1062. 10.1016/j.wneu.2019.01.243 30790729

[B12] Frahm J. Voit D. Uecker M. (2019). Real-Time Magnetic Resonance Imaging. Invest. Radiol. 54, 757–766. 10.1097/RLI.0000000000000584 31261294

[B13] Hata N. Song S.-E. Olubiyi O. Arimitsu Y. Fujimoto K. Kato T. (2016). Body-mounted Robotic Instrument Guide for Image-Guided Cryotherapy of Renal Cancer. Med. Phys. 43, 843–853. 10.1118/1.4939875 26843245 PMC4723400

[B14] He Z. Dong Z. Fang G. Ho J. D.-L. Cheung C.-L. Chang H.-C. (2020). Design of a Percutaneous MRI-Guided Needle Robot with Soft Fluid-Driven Actuator. IEEE Robot. Autom. Lett. 5, 2100–2107. 10.1109/LRA.2020.2969929

[B15] Hennersperger C. Fuerst B. Virga S. Zettinig O. Frisch B. Neff T. (2017). Towards MRI-Based Autonomous Robotic US Acquisitions: A First Feasibility Study. IEEE Trans. Med. Imaging 36, 538–548. 10.1109/TMI.2016.2620723 27831861

[B16] Hiraki T. Kamegawa T. Matsuno T. Sakurai J. Kirita Y. Matsuura R. (2017). Robotically Driven CT-guided Needle Insertion: Preliminary Results in Phantom and Animal Experiments. Radiology 285, 454–461. 10.1148/radiol.2017162856 28604237

[B17] Huang Q. Lan J. Li X. (2019). Robotic Arm Based Automatic Ultrasound Scanning for Three-Dimensional Imaging. IEEE Trans. Ind. Inf. 15, 1173–1182. 10.1109/TII.2018.2871864

[B18] Hungr N. Bricault I. Cinquin P. Fouard C. (2016). Design and Validation of a CT- and MRI-Guided Robot for Percutaneous Needle Procedures. IEEE Trans. Robot. 32, 973–987. 10.1109/TRO.2016.2588884

[B19] Hunsche S. Sauner D. Majdoub F. E. Neudorfer C. Poggenborg J. Goßmann A. (2017). Intensity-based 2D 3D Registration for lead Localization in Robot Guided Deep Brain Stimulation. Phys. Med. Biol. 62, 2417–2426. 10.1088/1361-6560/aa5ecd 28169225

[B20] Khan A. Meyers J. E. Yavorek S. O'Connor T. E. Siasios I. Mullin J. P. (2019). Comparing Next-Generation Robotic Technology with 3-Dimensional Computed Tomography Navigation Technology for the Insertion of Posterior Pedicle Screws. World Neurosurg. 123, e474–e481. 10.1016/j.wneu.2018.11.190 30500593

[B21] Kronreif G. Fürst M. Kettenbach J. Figl M. Hanel R. (2003). Robotic Guidance for Percutaneous Interventions. Adv. Robotics 17, 541–560. 10.1163/15685530360675532

[B22] Kundrat D. Dagnino G. Kwok T. M. Y. Abdelaziz M. E. M. K. Chi W. Nguyen A. (2021). An MR-Safe Endovascular Robotic Platform: Design, Control, and *Ex-Vivo* Evaluation. IEEE Trans. Biomed. Eng. 1, 1. 10.1109/TBME.2021.3065146 33705306

[B23] Lee K.-H. Fu K. C. D. Guo Z. Dong Z. Leong M. C. W. Cheung C.-L. (2018). MR Safe Robotic Manipulator for MRI-Guided Intracardiac Catheterization. Ieee/asme Trans. Mechatron. 23, 586–595. 10.1109/TMECH.2018.2801787

[B24] Lefranc M. Peltier J. (2015). Accuracy of Thoracolumbar Transpedicular and Vertebral Body Percutaneous Screw Placement: Coupling the Rosa Spine Robot with Intraoperative Flat-Panel CT Guidance-A Cadaver Study. J. Robotic Surg. 9, 331–338. 10.1007/s11701-015-0536-x 26530846

[B25] Li G. Patel N. A. Melzer A. Sharma K. Iordachita I. Cleary K. (2020). MRI-guided Lumbar Spinal Injections with Body-Mounted Robotic System: Cadaver Studies. Minimally Invasive Ther. Allied Tech. 0, 1–9. 10.1080/13645706.2020.1799017 PMC7855543 32729771

[B26] Lindenroth L. Soor A. Hutchinson J. Shafi A. Back J. Rhode K. (2017). Design of a Soft, Parallel End-Effector Applied to Robot-Guided Ultrasound Interventions. in 2017 IEEE/RSJ Int. Conf. Intell. Robots Syst. (Iros). 3716–3721. 10.1109/IROS.2017.8206219

[B27] Lonner J. H. Smith J. R. Picard F. Hamlin B. Rowe P. J. Riches P. E. (2015). High Degree of Accuracy of a Novel Image-free Handheld Robot for Unicondylar Knee Arthroplasty in a Cadaveric Study. Clin. Orthopaedics Relat. Research® 473, 206–212. 10.1007/s11999-014-3764-x PMC4390929 25002214

[B28] Marchand R. C. Sodhi N. Bhowmik-Stoker M. Scholl L. Condrey C. Khlopas A. (2019). Does the Robotic Arm and Preoperative CT Planning Help with 3D Intraoperative Total Knee Arthroplasty Planning? J. Knee Surg. 32, 742–749. 10.1055/s-0038-1668122 30112739

[B29] Mathiassen K. Fjellin J. E. Glette K. Hol P. K. Elle O. J. (2016). An Ultrasound Robotic System Using the Commercial Robot UR5. Front. Robot. AI 3. 10.3389/frobt.2016.00001

[B30] Melzer A. Gutmann B. Remmele T. Wolf R. Lukoscheck A. Bock M. (2008). INNOMOTION for Percutaneous Image-Guided Interventions. IEEE Eng. Med. Biol. Mag. 27, 66–73. 10.1109/EMB.2007.910274 18519184

[B31] Moreira P. van de Steeg G. Krabben T. Zandman J. Hekman E. E. G. van der Heijden F. (2017). The MIRIAM Robot: A Novel Robotic System for MR-Guided Needle Insertion in the Prostate. J. Med. Robot. Res. 02, 1750006. 10.1142/S2424905X17500064

[B32] O’Connor T. E. O’Hehir M. M. Khan A. Mao J. Z. Levy L. C. Mullin J. P. (2021). Mazor X Stealth Robotic Technology: A Technical Note. World Neurosurg. 145, 435–442. 10.1016/j.wneu.2020.10.010 33059080

[B33] Patel N. Yan J. Li G. Monfaredi R. Priba L. Donald-Simpson H. (2021). Body-Mounted Robotic System for MRI-Guided Shoulder Arthrography: Cadaver and Clinical Workflow Studies. Front. Robot. AI 8. 10.3389/frobt.2021.667121 PMC8141739 34041276

[B34] Ponzio D. Y. Lonner J. H. (2015). Preoperative Mapping in Unicompartmental Knee Arthroplasty Using Computed Tomography Scans Is Associated with Radiation Exposure and Carries High Cost. The J. Arthroplasty 30, 964–967. 10.1016/j.arth.2014.10.039 25840872

[B35] Seitz P. K. Baumann B. Johnen W. Lissek C. Seidel J. Bendl R. (2020). Development of a Robot-Assisted Ultrasound-Guided Radiation Therapy (USgRT). Int. J. CARS 15, 491–501. 10.1007/s11548-019-02104-y 31832907

[B36] Sensakovic W. F. O’Dell M. C. Agha A. Woo R. Varich L. (2017). CT Radiation Dose Reduction in Robot-Assisted Pediatric Spinal Surgery. Spine 42, E417–E424. 10.1097/BRS.0000000000001846 27513224

[B37] Shahriari N. Heerink W. van Katwijk T. Hekman E. Oudkerk M. Misra S. (2017). Computed Tomography (CT)-compatible Remote center of Motion Needle Steering Robot: Fusing CT Images and Electromagnetic Sensor Data. Med. Eng. Phys. 45, 71–77. 10.1016/j.medengphy.2017.04.009 28512000

[B38] Smakic A. Rathmann N. Kostrzewa M. Schönberg S. O. Weiß C. Diehl S. J. (2018). Performance of a Robotic Assistance Device in Computed Tomography-Guided Percutaneous Diagnostic and Therapeutic Procedures. Cardiovasc. Intervent Radiol. 41, 639–644. 10.1007/s00270-017-1841-8 29159685

[B39] Spyrantis A. Cattani A. Strzelczyk A. Rosenow F. Seifert V. Freiman T. M. (2018). Robot-guided Stereoelectroencephalography without a Computed Tomography Scan for Referencing: Analysis of Accuracy. Int. J. Med. Robotics Comput. Assist. Surg. 14, e1888. 10.1002/rcs.1888 29316270

[B40] Stoianovici D. Cleary K. Patriciu A. Mazilu D. Stanimir A. Craciunoiu N. (2003). AcuBot: a Robot for Radiological Interventions. IEEE Trans. Robot. Automat. 19, 927–930. 10.1109/TRA.2003.817072

[B41] Unger M. Berger J. Gerold B. Melzer A. (2020). Robot-assisted Ultrasound-Guided Tracking of Anatomical Structures for the Application of Focused Ultrasound. Curr. Dir. Biomed. Eng. 6, 123–126. 10.1515/cdbme-2020-3032

[B42] Vadera S. Chan A. Lo T. Gill A. Morenkova A. Phielipp N. M. (2017). Frameless Stereotactic Robot-Assisted Subthalamic Nucleus Deep Brain Stimulation: Case Report. World Neurosurg. 97, e11–762.e14 10.1016/j.wneu.2015.11.009 26585721

[B43] Virga S. Zettinig O. Esposito M. Pfister K. Frisch B. Neff T. (2016). Automatic Force-Compliant Robotic Ultrasound Screening of Abdominal Aortic Aneurysms. in 2016 IEEE/RSJ Int. Conf. Intell. Robots Syst. (Iros). 508–513 10.1109/IROS.2016.7759101

[B44] Won H. J. Kim N. Kim G. B. Seo J. B. Kim H. (2017). Validation of a CT-guided Intervention Robot for Biopsy and Radiofrequency Ablation: Experimental Study with an Abdominal Phantom. Diagn. Interv. Radiol. 23, 233–237. 10.5152/dir.2017.16422 28287073 PMC5411006

[B45] Yang Y. Jiang S. Yang Z. Yuan W. Dou H. Wang W. (2017). Design and Analysis of a Tendon-Based Computed Tomography-Compatible Robot with Remote center of Motion for Lung Biopsy. Proc. Inst. Mech. Eng. H 231, 286–298. 10.1177/0954411917690763 28195001

[B46] Zaffino P. Moccia S. De Momi E. Spadea M. F. (2020). A Review on Advances in Intra-operative Imaging for Surgery and Therapy: Imagining the Operating Room of the Future. Ann. Biomed. Eng. 48, 2171–2191. 10.1007/s10439-020-02553-6 32601951

